# Supplementary material for: BayFlux: A Bayesian method to quantify metabolic Fluxes and their uncertainty at the genome scale
Source: PLoS Comput Biol. 2023 Nov 10;19(11):e1011111. doi: 10.1371/journal.pcbi.1011111 (PMC10664898; doi:10.1371/journal.pcbi.1011111)
Supplement: S1 Text — (PDF) [file pcbi.1011111.s001.pdf]

Supporting Information for BayFlux: A *Bay*esian method to quantify metabolic *Flux*es and their uncertainty at the genome scale

Tyler W. H. Backman[1,2], Christina Schenk[1,3,4], Tijana Radivojevic[1,2,4], David Ando[1,2], Jahnavi Singh[11], Jeffrey J. Czajka[5], Zak Costello[1,2,4], Jay D. Keasling[1,2,6,7,8,9,10], Yinjie Tang[5], Elena Akhmatskaya[1,3,12], Hector Garcia Martin[1,2,3,4*]

1 Biological Systems and Engineering Division, Lawrence Berkeley National Laboratory, Berkeley, California, USA
2 Biofuels and Bioproducts Division, Joint BioEnergy Institute, Emeryville, California, USA
3 BCAM, Basque Center for Applied Mathematics, Bilbao, Spain
4 DOE Agile BioFoundry, Emeryville, California, USA
5 Department of Energy, Environmental and Chemical Engineering, Washington University in St. Louis, St. Louis, Missouri, USA
6 Department of Chemical and Biomolecular Engineering, University of California, Berkeley, California, USA
7 Department of Bioengineering, University of California, Berkeley, California, USA
8 QB3 Institute, University of California, Berkeley, California, USA
9 Novo Nordisk Foundation Center for Biosustainability, Technical University of Denmark, Copenhagen, Denmark
10 Center for Synthetic Biochemistry, Institute for Synthetic Biology, Shenzhen Institutes for Advanced Technologies, Shenzhen, China
11 Department of Electrical Engineering and Computer Sciences, University of California, Berkeley, California, USA
12 IKERBASQUE, Basque Foundation for Science, Bilbao, Spain
13 Center for Synthetic Biochemistry, Institute for Synthetic Biology, Shenzhen Institutes for Advanced Technologies, Shenzhen, China

* hgmartin@lbl.gov

Glossary of terms

Term	Description
Cyclic flux	Simultaneous forward and backwards flux within a single (reversible) metabolic reaction. Quantified as $forward\ flux - backwards\ flux$. Sometimes referred to as an "exchange flux" in ^{13}C MFA literature [1], but we avoid using the word exchange to avoid confusion with extracellular exchange fluxes.
(Extracellular) exchange flux	Flux of reactions which exchange metabolites outside of the organism or cell, for example the uptake of an extracellular nutrient. Often, in a liquid cell culture, this can be measured directly by a change in extracellular concentration over time.
Edge points	Points on the edge of the feasible flux polytope, used by the ACHR (Artificial Centering Hit and Run algorithm) [2] as reference points to determine movement directions for the sampler. These are typically the maximum and minimum fluxes for each reaction in the metabolic model, as determined through linear optimization. Often called "warm-up points" in ACHR literature, we use the word 'edge' to avoid confusion with MCMC (Markov Chain Monte Carlo) warm-up samples.
Warm-up samples	Samples collected during the initial period of a MCMC (Markov Chain Monte Carlo) run, during which the sampler is unlikely to have yet reached a stationary distribution. These samples are typically discarded when analyzing the posterior probability distribution.

References

1. Wiechert W. The thermodynamic meaning of metabolic exchange fluxes. Biophysical Journal. 2007;93(6):2255–2264. doi:10.1529/biophysj.106.099895.

2. Kaufman DE, Smith RL. Direction Choice for Accelerated Convergence in Hit-and-Run Sampling. Operations Research. 1998;46(1):84–95.

3. Buescher JM, Antoniewicz MR, Boros LG, Burgess SC, Brunengraber H, Clish CB, et al. A roadmap for interpreting 13C metabolite labeling patterns from cells. Current opinion in biotechnology. 2015;34:189–201.

4. Toya Y, Ishii N, Nakahigashi K, Hirasawa T, Soga T, Tomita M, et al. 13C-metabolic flux analysis for batch culture of Escherichia coli and its pyk and pgi gene knockout mutants based on mass isotopomer distribution of intracellular metabolites. Biotechnology progress. 2010;26(4):975–992.

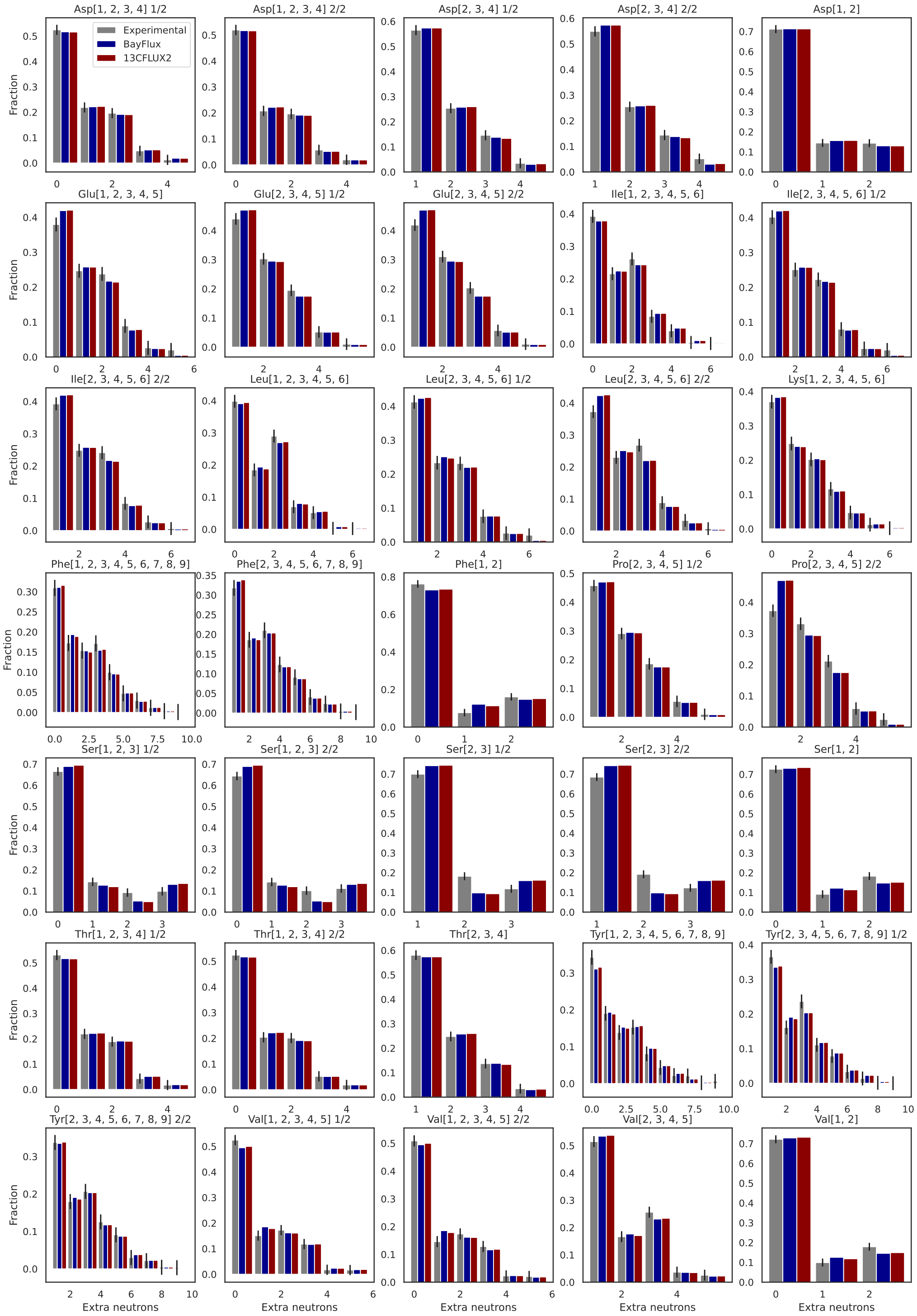

Fig A. Fit labeling patterns are the same for both approaches, BayFlux and 13CFLUX2. Mass distributions vectors (MDVs) for experimental data (grey), the fits for the best sample for BayFlux (dark blue, ten million samples), and the fits for 13CFLUX2 (red) are closer together than the standard deviation of the experimental error. Units for horizontal axis are the number of extra neutrons in the measured metabolite [3].

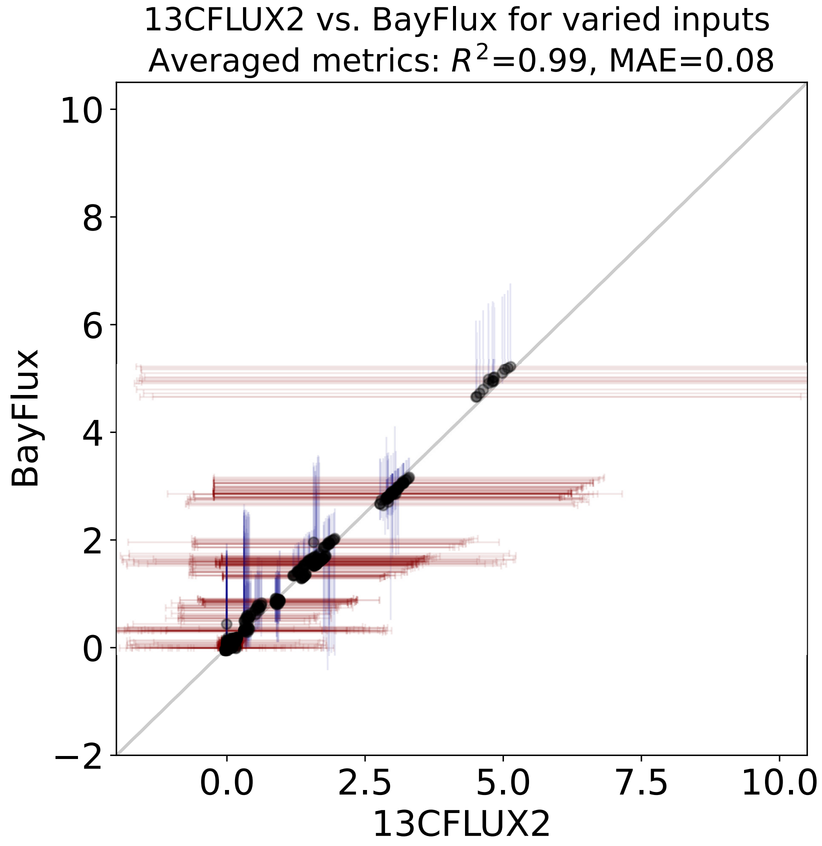

Fig B. Flux results for BayFlux and 13CFLUX2 are essentially the same for fifteen different inputs. The best BayFlux sample (dark blue, ten million samples, y axis) and its credible interval overlap with the 13CFLUX2 best fit (in red, x axis) and its confidence interval for all fifteen different inputs and fluxes. The fifteen different inputs were obtained by randomly changing the exchange fluxes, but keeping the same labeling profiles for the metabolites. Both axes are in units of mmol/gDW/h. We set the 13CFLUX2 confidence bounds that could not be determined to the overall maximum BayFlux bounds for comparison purposes.

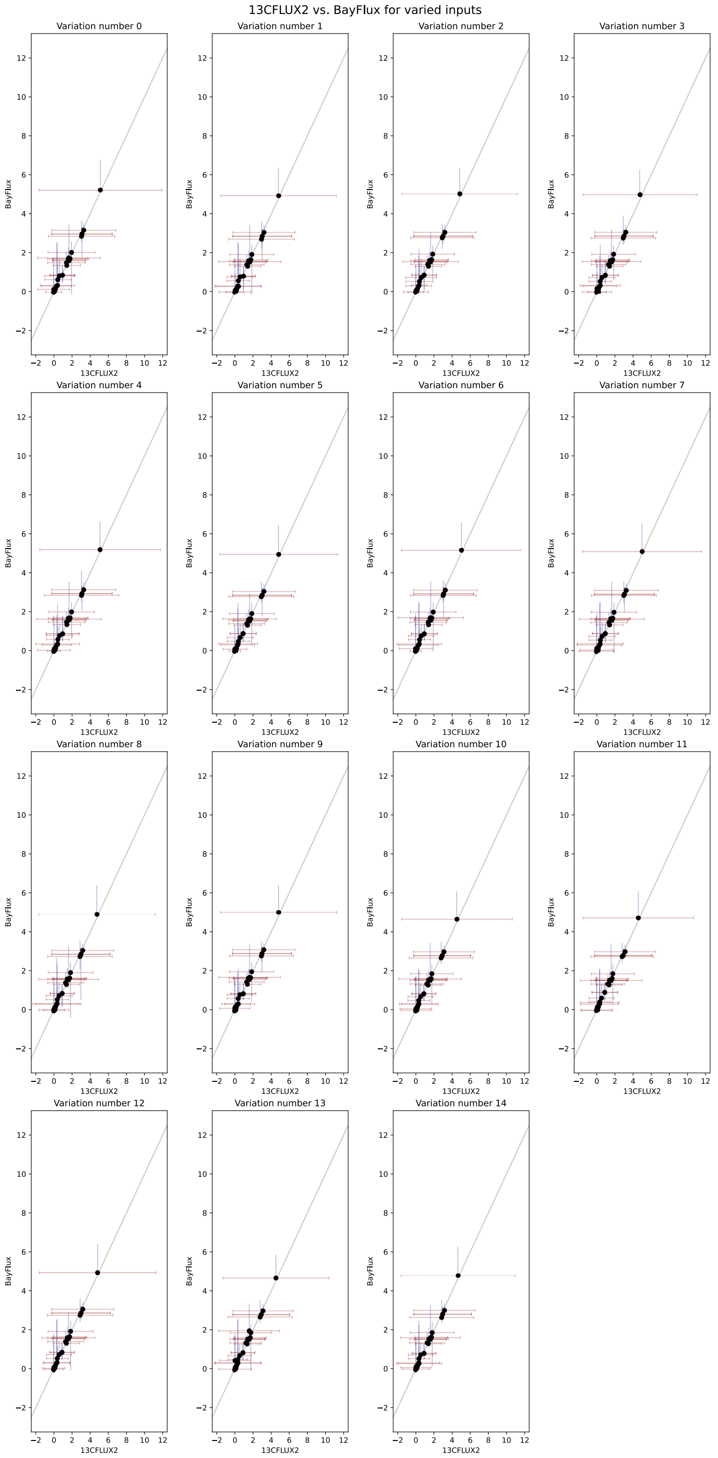

Fig C. 13CFLUX2 vs. BayFlux for 15 different inputs: Best Sample out of 10 million from BayFlux (in dark blue) vs. 13CFLUX2 (in red). We set the 13CFLUX2 confidence bounds that could not be determined to the overall maximum BAYFLUX bounds for comparison purposes.

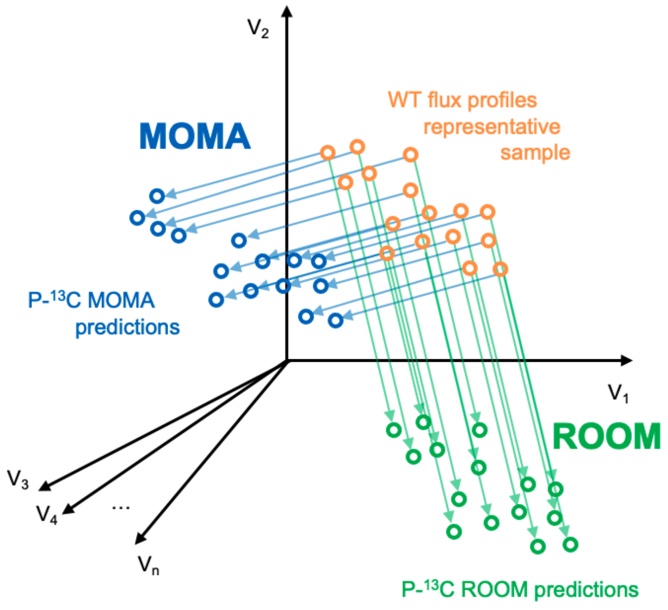

Fig D. P-^{13}C MOMA and P-^{13}C ROOM work by first computing the WT flux profile distribution using BayFlux (in orange). Each dot corresponds to a flux profile (*i.e.* reaction fluxes for all reactions in a metabolic model) in the flux phase space. A representative set of flux profiles for this base flux profile distribution is obtained through sampling (orange dots). For each of these flux profiles, a new flux profile is computed by using MOMA (in blue) or ROOM (in green) to predict the resulting flux after a knockout. The fact that BayFlux produces a flux profile distribution results in MOMA and ROOM predicting distributions of flux profiles.

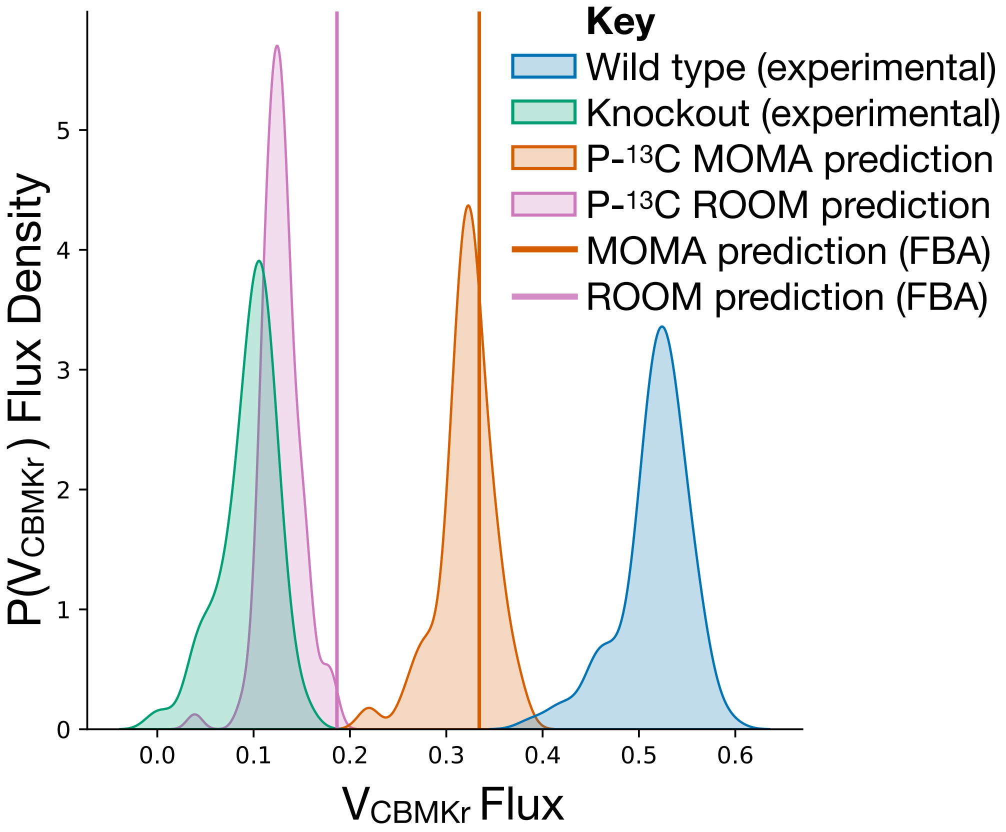

Fig E. Using BayFlux to predict gene knockout genome-scale flux distributions. We employ P-^{13}C MOMA and P-^{13}C ROOM (brown and violet respectively) to predict the flux distributions for the *pgi* gene knockout (green, Toya *et al.* pgi16h data [4], computed using BayFlux) by leveraging the wild type flux distribution (blue, Toya *et al.* wt5h data [4], computed using BayFlux), and compare to FBA based MOMA and ROOM methods (brown and violet vertical lines respectively). For the CBMKr (Carbamate kinase) flux, the probability distributions from P-^{13}C ROOM is much closer to the experimentally obtained probability distribution for the knockout (computed from experimental data using BayFlux) indicating a more accurate prediction. The probability distribution from P-^{13}C MOMA is only marginally closer to the experimentally obtained probability distribution for the knockout since the peak of the distribution is a bit closer than the single value FBA MOMA prediction. Here, we show only one reaction as an illustration of the approach, whereas the true demonstration that the predictions are better must involve all fluxes and is shown in Fig. 7 in the main text.
